# Inflammation-induced alterations in maternal-fetal Heme Oxygenase (HO) are associated with sustained innate immune cell dysregulation in mouse offspring

**DOI:** 10.1371/journal.pone.0252642

**Published:** 2021-06-04

**Authors:** Maide Ozen, Hui Zhao, Flora Kalish, Yang Yang, Lauren L. Jantzie, Ronald J. Wong, David K. Stevenson

**Affiliations:** 1 Department of Pediatrics, Division of Neonatal-Perinatal Medicine, Johns Hopkins University School of Medicine, Baltimore, Maryland, United States of America; 2 Department of Pediatrics, Division of Neonatal and Developmental Medicine, Stanford University School of Medicine, Stanford, California, United States of America; 3 Department of Genetics, Stanford University School of Medicine, Stanford, California, United States of America; University of Wisconsin - Madison, School of Veterinary Medicine, UNITED STATES

## Abstract

Heme oxygenase-1 (HO-1) is an evolutionarily conserved stress response enzyme and important in pregnancy maintenance, fetal and neonatal outcomes, and a variety of pathologic conditions. Here, we investigated the effects of an exposure to systemic inflammation late in gestation [embryonic day (E)15.5] on wild-type (Wt) and HO-1 heterozygous (Het, HO-1^+/-^) mothers, fetuses, and offspring. We show that alterations in fetal liver and spleen HO homeostasis during inflammation late in gestation can lead to a sustained dysregulation of innate immune cell populations and intracellular myeloid HO-1 expression in the spleen through young adolescence [postnatal day 25] in mice.

## Introduction

Normal pregnancy is an immunotolerant state [1]. However, infection and/or inflammation during pregnancy can have devastating consequences for a mother, fetus, and newborn [1]. Adverse effects include the loss of maternal immunotolerance to the fetus, which can lead to spontaneous abortions, stillbirths, or preterm deliveries, as well as to a dysregulation of the fetal and neonatal immune systems and subsequent organ dysfunction [1, 2]. Even low-grade subclinical infections during pregnancy may result in long-lasting complications in the offspring, such as a sustained proinflammatory disposition [3, 4].

Heme-oxygenase-1 (HO-1), the rate-limiting enzyme in the heme degradation pathway, is the inducible HO isozyme known to regulate a number of important physiologic functions [1, 5, 6], such as cytoprotection, mitochondrial activity, and antioxidant defense [7]. It is also central to immune homeostasis [1, 2]. As such, it is evolutionarily conserved across many species [8, 9]. Importantly, HO-1 is crucial for the establishment and maintenance of a normal pregnancy [1, 10]. In fact, we have previously shown that a partial or total deficiency of HO-1 could lead to abnormalities in myeloid cell infiltration and placental vascular development [11, 12]. HO-1 is also involved in the regulation of the innate immune system, specifically neutrophils, monocytes, macrophages (Mϕ), and dendritic cells (DCs) [13], and in the modulation of adaptive immune system responses [14].

By orchestrating a multitude of immune adaptations during each trimester of pregnancy, HO-1 is integral to pregnancy success starting from placentation, spiral artery remodeling, vascular changes, and maternal acceptance of the semi-allogeneic fetus [13]. A perturbed HO-1 state (such as in a deficiency of HO-1) can result in abnormal placentation, abortions, or preeclampsia, which can be rescued pharmacologically via its upregulation or immunomodulation as shown in animal models [12, 13, 15]. Although these strategies may confer protection from pregnancy complications such as fetal loss [15], the uncontrolled, prolonged, and/or sustained induction of HO-1 can also be detrimental and contribute to a low-grade inflammatory state [16–19]. For instance, a sustained increase in HO-1 leads to cellular cytotoxicity; whereas, a low-level induction is cytoprotective [16]. Furthermore, emerging data support that an excessive upregulation of HO-1 might be harmful through iron-dependent lipid peroxidation and results in cell death and inflammation [18, 20]. The exact mechanism however by which the induction of HO-1 causes this inflammatory response needs further exploration. The basal expression level of HO-1 is believed to determine protective properties in the absence of a stressor [21, 22]. Because pregnancy is a known stressor, maintaining a delicate balance of HO-1 upregulation in response to pregnancy stress or inflammation, and convalescing to a pre-pregnancy or pre-disease baseline is crucial to prevent potential sequelae.

In this study, we hypothesized that subclinical maternal inflammation in late pregnancy can upset the maternal-fetal HO-1 balance, adversely affect and reprogram the neonatal innate immune system, and consequently disrupt the development of a normal immune system in offspring.

## Materials and methods

### Animals

Timed pregnancies were established by breeding adult wild-type (Wt HO-1^+/+^), FVB females (6- to 8-wks old) with adult Wt FVB males (10- to 12-wks old) or HO-1 heterozygote (HO-1^+/-^, Het) females (6- to 8-wks old) with adult Het males (10- to 12-wks old) overnight at a ratio of 2 females with 1 male. A subset of age- and genotype-matched, non-pregnant (NP) females served as controls. Our Het colony was originally established by backcrossing Wt with HO-1 knockout (HO-1^-/-^, KO, original source from Poss and Tonegawa [23]) mice for more than 6 generations. In our experience, Het pregnancies normally yield ~70% Het, 25% Wt, and 2% to 5% KO pups [12, 23]. No Wt pups delivered from Het dams were used in Wt studies. Pregnancies were confirmed by the presence of a vaginal plug at E0.5 and/or evidence of maternal weight gain from E9.5 to E15.5. Pregnant dams were stratified to the lipopolysaccharide (LPS) or vehicle (normal saline) group at E15.5. Maternal health and weight gain were monitored daily until delivery. After delivery, pups were housed only with their birth mothers. Post-birth, the health of pups was monitored daily for the first week and weekly thereafter using weight gain as an index of general well-being [2]. Animals were euthanized by CO_2_ inhalation and all efforts were made to minimize suffering in accordance with our approved animal protocol and no analgesics were required. All mice were housed in Innovive recyclable individually ventilated (IVC) cages (San Diego, CA) in a 25 ± 1°C room with a 12 h light:12 h dark cycle starting at 0700 and allowed food (Teklad Rodent Diet #2018, Envigo, Hayward, CA) and pre-filled acidified drinking water (Aquavive^®^, Innovive) *ad libitum*. All experiments were conducted at Stanford University and were approved by the Stanford University Administrative Panel on Laboratory Animal Care (APLAC #12884, #6691). The health status of the mouse colonies was continuously assessed by the Veterinary Service Center at Stanford University Department of Comparative Medicine by routinely monitoring for a number of murine viruses, bacteria, and parasites (minute virus of mice [MVM], mouse hepatitis virus [MHV], mouse rotavirus [EDIM], Theiler’s murine encephalomyelitis virus [TMEV], Sendai virus, mouse adenovirus 1 and 2 [MAdV1 and 2], Ectromelia [mousepox, ECTV], lymphocytic choriomeningitis virus [LCMV] pneumonia virus of mice [PVM], respiratory enteric virus 3 [Reovirus 3], Mycoplasma pulmonis, ectoparasites, including fur mites, and pinworms. During the entire course of these studies, our mouse colonies tested negative for any of these pathogens.

### Genotyping

Genomic DNA was obtained from tail clippings to identify HO-1 Het and KO mice as previously described [5]. In brief, 2 sets of primers designed for the Wt, Het, and KO genotypes were used. Wt (510 bp) and Het (390 bp) bands were determined by gel electrophoresis [12].

### Study design

We conducted this study using our established late gestational inflammation model [2]. In brief, lipopolysaccharide (LPS at 90 μg/kg [LPS90] *Escherichia coli*, O55:B5, L5418, ready-made solution, Sigma-Aldrich, St. Louis, MO) was administered intraperitoneal (i.p.) to pregnant Wt mice at E15.5, which corresponds to the second trimester of a human pregnancy. Control dams received an equal volume of vehicle (Veh, normal saline) i.p. We were able to establish that LPS90 was associated with placental pathology, including an increased percentage of laminar decidua necrosis at the decidua-spongiotrophoblastic region at E16.5, a decrease in pup survival (70%) at delivery and persistent adaptive immune system alterations in Wt and Het offspring at postnatal age (PN) 14 and 21 [2].

**Fig 1:**
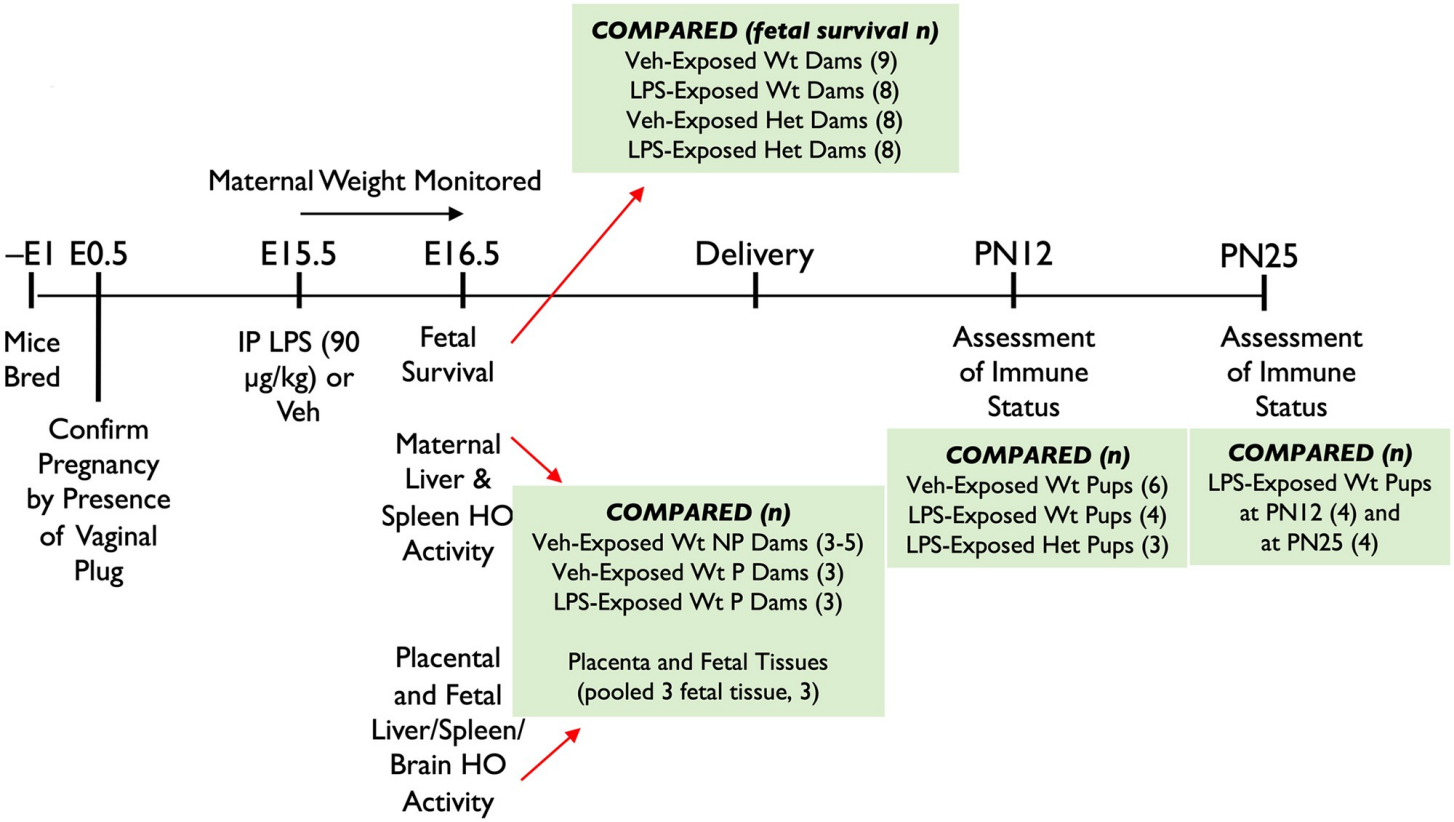
Late gestation inflammation model. LPS, lipopolysaccharide; NP, non-pregnant; Veh, vehicle; Wt, wild-type; Het, HO-1 heterozygote; PN, post-natal; i.p., intraperitoneal; HO, heme oxygenase; E, embryonic age.

In this study, we determined the acute effects of late gestational inflammation 24 hrs (at E16.5) after exposure to LPS90. We then investigated whether an exposure to LPS90 results in fetoplacental inflammation and innate immune system reprogramming in the offspring at PN12 and PN25, which may have preceded our previously reported adaptive immune system changes using this model (Fig 1).

### Assessment of maternal well-being

Health of dams treated with LPS at E15.5 was assessed at 24 hrs (at E16.5) post-LPS90 administration by monitoring the presence of any acute weight loss as described by Kim et al [24]. Weights were compared with those of Veh-treated, age-matched pregnant mice (controls).

### Assessment of fetal viability

To assess fetal viability, the number of total (viable plus resorbed) fetuses were recorded at E16.5 and the percentages of live and resorbed fetuses per pregnancy were calculated. Resorbed fetuses were defined when necrotic/hemorrhagic tissue without a definitive delineation between the fetus and the placenta was observed within the gestational sac [15]. A total of 9 Wt control, 8 Het control, 8 Wt LPS90 and 8 Het LPS90 dams were used for fetal viability studies.

### Measurements of total HO enzyme activity

For HO activity measurements, tissues were processed and activity was measured by gas chromatography as previously described [25]. We focused on the liver and spleen because they highly express HO-1 and are involved in the development of the fetal immune system. Baseline liver and spleen HO activities were determined in age-matched NP untreated Wt females. To assess the effect of LPS on liver and spleen HO activities, tissues were harvested from age-matched LPS-treated Wt P, Veh-treated pregnant (P), and untreated NP females. In addition, HO activity in placentas, fetal liver/spleen pairs, and fetal brains (pooled 3 fetal tissue/litter into one sample and a total of three samples coming from 9 fetal tissues were used for this experiment) harvested 24 hrs after maternal exposure to LPS were compared with untreated gestational age (GA)- and genotype-matched control tissues. Pups from a minimum of 2 litters or more were used for each study in order to eliminate any litter biases. Because at E16.5 fetal tissues are very fragile and minute, we decided *a priori* to include liver and spleen together as a pooled sample and irrespective of sex (as identification was not possible).

### Multi-parameter flow cytometry

After treatment with LPS90 at E15.5, pregnant Wt and Het dams were let to deliver. Spleens from PN12 and PN25 offspring were dissociated using a Gentle MACS Dissociator (Miltenyi Biotec, Auburn, CA), passed through a 70-μm nylon mesh cell strainer (BD Falcon, BD BioSciences, San Jose, CA), after determining viability by trypan blue exclusion cell suspensions were stained [2].

Suspensions were then incubated with viability dye in phosphate buffered saline (PBS) for 30 mins at 4°C, followed by Fc block with CD16/32 and staining (1:100) for myeloid cell surface markers with: fixable viability dye eFluor 506 (Cat No. 65086614), CD45-PerCP-Cy5.5 (Cat No. 45045182), CD11c-PE-Cy7 (Cat No. 25011482), CD11b-PE (Cat No. 12011282), Ly6G Biotin (Cat No. 127603), Streptavidin qdot 605 (Cat No. Q10103MP), Ly6C-Alexa 700 (Cat No. 128024), MHC II-APC (Cat No. 17532181), CD19-Pacific Blue (Cat No. 115523), CD3-γΔTCR (Cat No. 100214), and CD16/32 (Cat No. 14016081) (all purchased from eBioScience, ThermoFisher Scientific, Waltham, MA) and HO-1 FITC (Cat No. ADI-OSA-111FI-F, Enzo Life Sciences, Inc. Farmingdale, NY). Cells were then fixed with X1 intracellular fixation buffer (Cat No. 88882400, stock X10 concentrate, eBioscience, ThermoFisher Scientific), permeabilized and stained intracellularly with HO-1 at the manufacturer’s recommended concentrations. Single stained compensation controls were utilized for all experiments. Flow cytometry was performed at the Stanford Shared FACS Facility on a LSR.II.2 (BD Biosciences). Debris and doublets were excluded by sequential gating on forward scatter height versus forward scatter area. Anti-CD19-Pacific Blue and anti-CD3-γΔTCR were used as dump channels. After gating on the viable cell population, we sequentially gated on CD45, CD11b, CD11c, MHC II, HO-1; CD45, Ly6G, HO-1; CD45, Ly6C, HO-1 for the myeloid cells. For HO-1, median fluorescence intensity (MFI) was calculated. A representative gating strategy is shown in S1 Fig. Data were analyzed using FlowJo 10 software (Tree Star, Ashland, OR).

### Statistical analyses

Data was compared using two-way ANOVAs with Tukey’s correction, 2-tailed Student’s t-tests with Welch correction when normally distributed by Shapiro-Wilk test and Kruskal-Wallis or Mann Whitney tests when not normally distributed using Prism software v8.0 (GraphPad, San Diego, CA). Survival differences were determined using log-rank tests. G-power analysis was used to calculate a statistical power of 99% for our sample size of 3 to 5 animals per group [26]. Data was expressed as median or mean ± SD. Results were deemed statistically significant when p < 0.05.

## Results

### Maternal systemic response following treatment with LPS at E15.5

24 hrs post LPS90-treatment, weight changes in pregnant Wt and Het dams were not significantly different from GA- and genotype-matched controls from E15.5 to E16.5 (S1 Table).

### Fetal viability following maternal treatment with LPS

**Fig 2:**
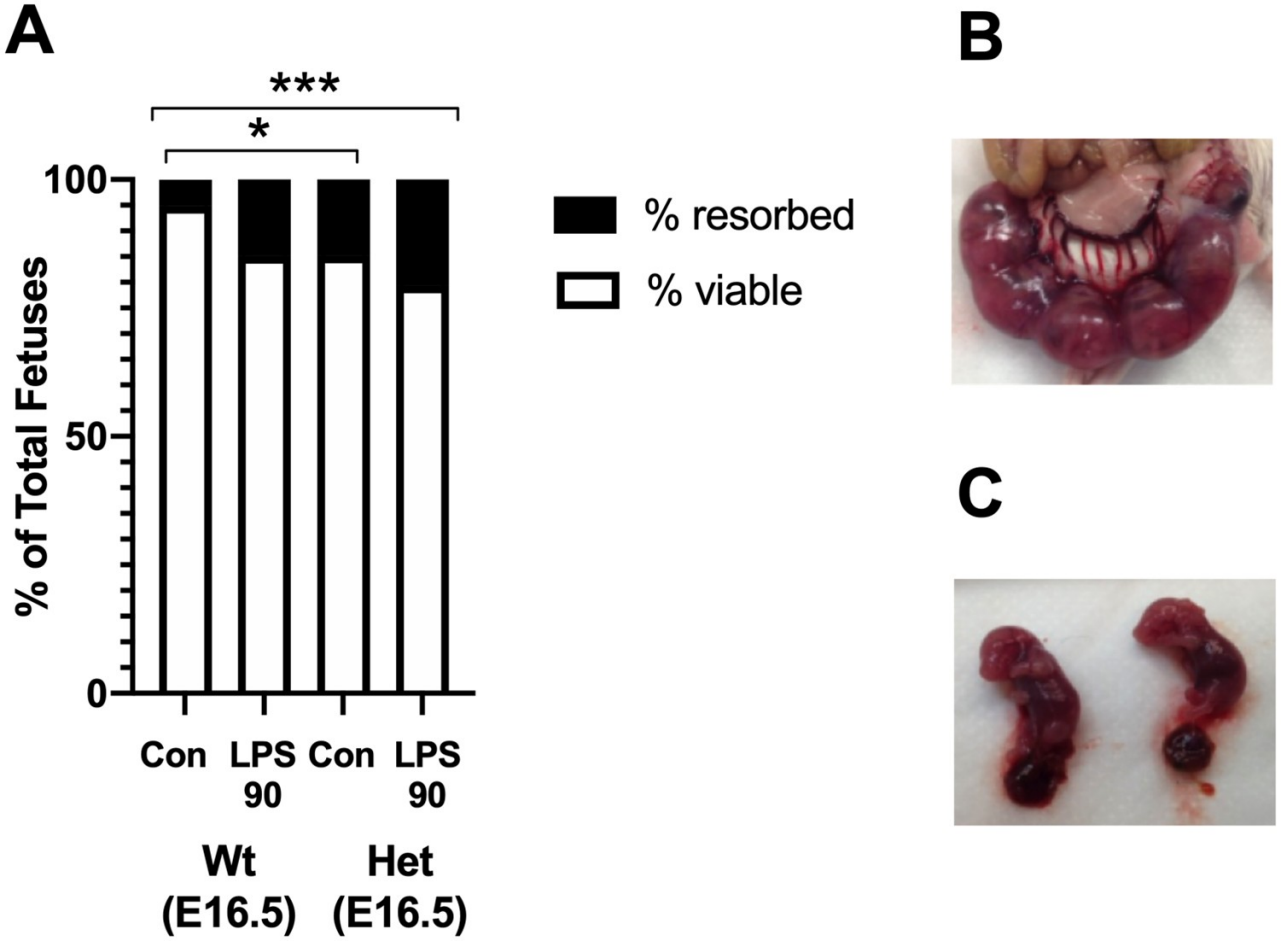
Fetal survival after LPS administration. (**A**) LPS at a dose of 90 ug/kg (LPS90) was administered i.p. to pregnant Wt or Het dams at E15.5 (control or Con, n = 9; LPS90, n = 8, sacrificed at E16.5). LPS90 was administered i.p. to pregnant- Het dams, at E15.5 (Con, n = 8; LPS90, n = 8, sacrificed at E16.5). Con were GA- and genotype-matched. For Wt, there was no significant decrease in percent live fetuses after LPS compared with genotype-matched controls. Fetal survival was significantly lower in untreated Het Con compared to untreated Wt Con at E16.5. Representative images taken 24 hrs after LPS90 administration of a (**B**) uterus and (**C**) fetuses from a Wt dam are shown. *p < 0.05, log-rank tests for viable versus resorbed.

Next, we set out to determine fetal viability 24 hrs (E16.5) after maternal exposure to LPS90 (Fig 2). The total number and percentages of live fetuses per litter were similar between LPS90-treated dams compared with pregnant Wt controls. Litter sizes for Wt control and Wt LPS90 ranged from 5 to 12 and 4 to 11 pups, respectively. Similar for Hets, the total number and percent live fetuses per litter were not different to that of GA-matched Het controls Litter sizes for Het control and Het LPS90 ranged from 5 to 9 and 4 to 11, respectively (Fig 2A).

In Het dams, the baseline survival of fetuses in the entire cohort of Het control pregnancies (61 total concepti, 10 resorbed concepti from 8 Het control dams, with survival proportion of 83.6 ± 4.7%) were significantly lower than Wt controls for entire cohort (88 total concepti, 4 resorbed concepti from 9 Wt Control dams, with survival proportion of 95.5 ± 2.2%) at E16.5. Survival for Het control concepti was significantly lower compared with Wt controls at E16.5 with log-rank test for the entire cohort (p = 0.015). LPS90-treated Wt dams (69 total concepti, 9 resorbed concepti from 8 Wt LPS90 dams, with survival proportion of 87.0 ± 4.1%), and Wt controls (88 total concepti, 4 resorbed concepti from 9 Wt Control dams, with survival proportion of 95.5 ± 2.2%) were similar at E16.5 for the entire cohort. Representative images of a Wt uterus and fetal conceptus harvested 24 hrs post-LPS90 treatment of Wt dams are shown on Fig 2B and 2C, respectively.

### HO activity in NP and pregnant Wt females following treatment with LPS

**Fig 3:**
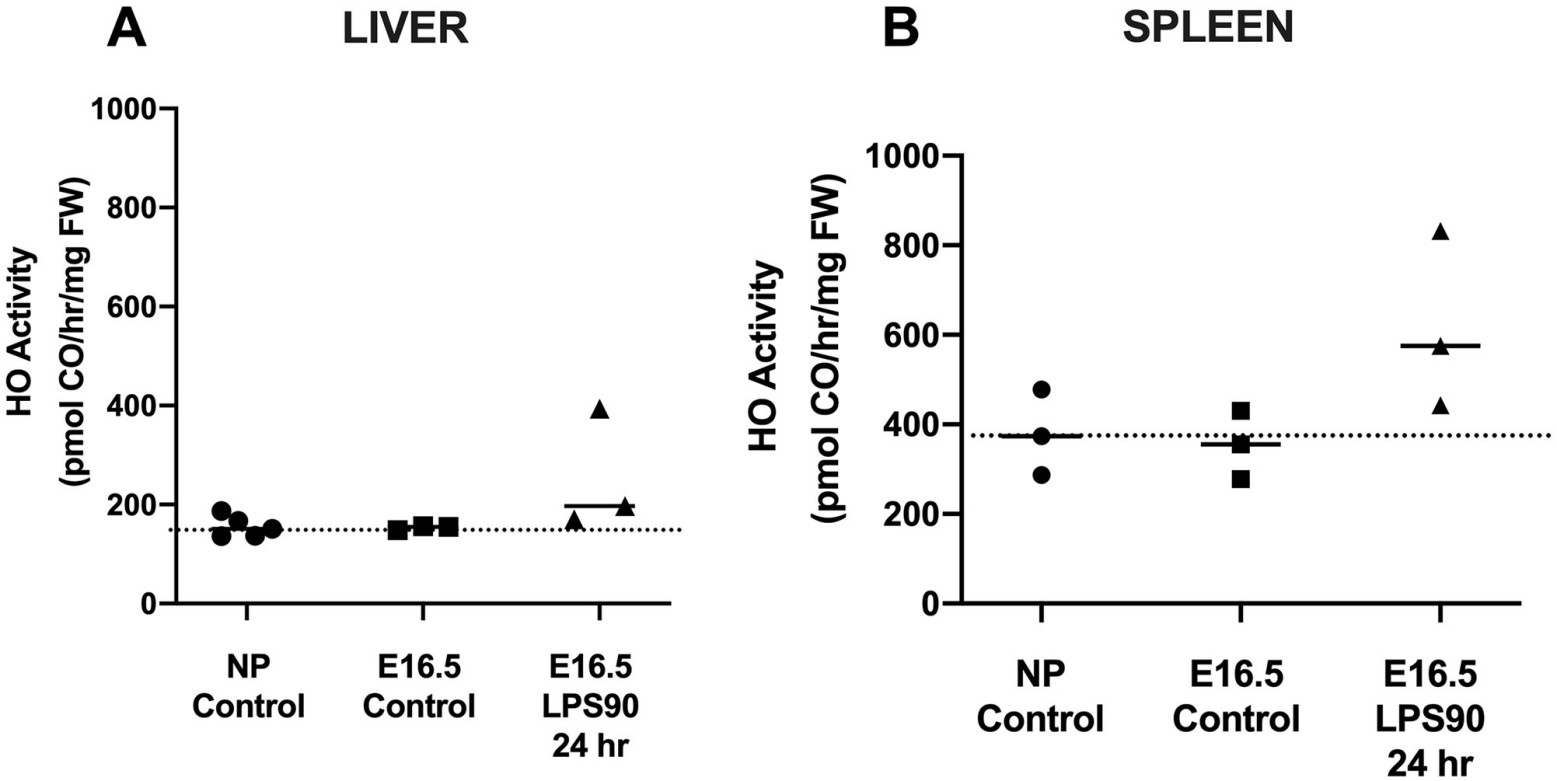
HO activity in liver and spleen of non-pregnant (NP) adult females and pregnant (P) dams following LPS at a dose of 90 ug/kg (LPS90). LPS was administered i.p. to pregnant Wt dams at E15.5 (Non-pregnant Control, n = 5; Control (vehicle), n = 3; LPS90, n = 3) and HO activity was measured 24 hrs later. Controls were matched by chronologic age, GA, and genotype. (**A**) Liver and (**B**) Spleen HO activity in NP females, pregnant control dams and LPS-treated dams were similar. FW: fresh weight. No statistical differences were found between any of the groups.

Next, we measured HO activity in NP Controls, pregnant controls at E16.5, and pregnant Wt dams 24 hrs following LPS90 treatment or at E16.5 (Fig 3). Liver HO activity was similar 24 hrs post-LPS90 (254 ± 122 pmol/hr/mg fresh weight [FW], n = 3) compared with pregnant GA-matched untreated Wt and NP controls (153 ± 3, n = 3, and 156 ± 22, n = 5, pmol/hr/mg FW, respectively) (Fig 3A). Spleen HO activity 24 hrs post-LPS90 was not significantly different from NP (617 ± 198 versus 380 ± 95.3 pmol/hr/mg FW, respectively) and GA-matched untreated controls (355 ± 76 pmol/hr/mg FW) (Fig 3B).

### Wt placental and fetal brain and liver and spleen pooled HO activities following LPS

**Fig 4:**
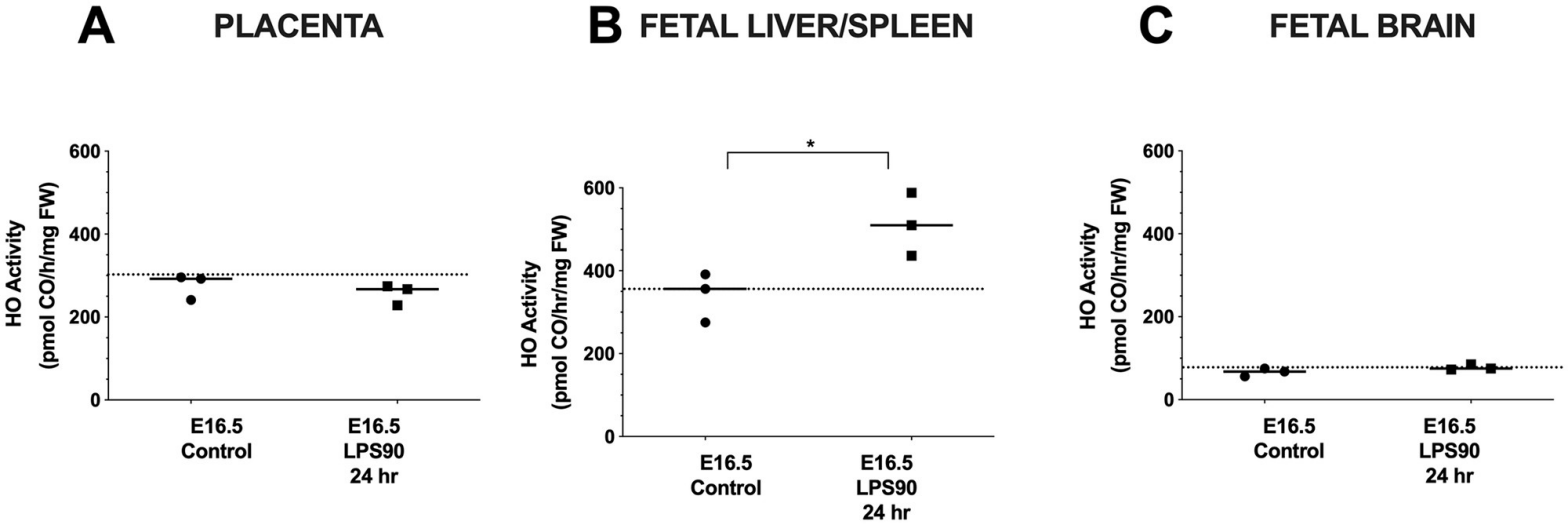
HO activity in placentas and fetal organs after LPS at a dose of 90 ug/kg (LPS90). LPS was administered i.p. to pregnant Wt dams, at E15.5 (Control (Veh), n = 3; LPS90, n = 3) and HO activity was measured 24 hrs later. (**A**) Placental HO activity was unchanged. (**B**) Fetal liver/spleen HO activity significantly increased 24 hrs after LPS exposure. p < 0.05, Welch’s t-test. (**C**) Fetal brain HO activity was unchanged when measured at E16.5. FW: fresh weight. (Data is normally distributed for placenta and fetal liver/spleen and brain.).

HO activity in Wt placentas was similar between LPS90 (257 ± 25 pmol/hr/mg FW, n = 3 [each pooled from 3 placentas]) and untreated controls (276 ± 31 pmol/hr/mg FW, n = 3 [each pooled from 3 placentas) (Fig 4A). However, Wt fetal liver/spleen HO activity significantly increased (511 ± 76 pmol/hr/mg FW, n = 3 [each pooled from 3 fetuses]) compared with control fetuses (341 ± 60 pmol/hr/mg FW, n = 3 [each pooled from 3 fetuses], p = 0.04) (Fig 4B). Fetal brains did not show significant increases in HO activity (78 ± 7 pmol/hr/mg FW, n = 3 [each pooled from 3 fetuses]) compared with GA-matched fetal controls (66 ± 10 pmol/hr/mg FW, n = 3 [each pooled from 3 fetuses]) (Fig 4C).

### Innate immune responses in offspring at PN12 after maternal treatment with LPS

The frequency of CD45^+^ cells (common leukocyte marker) were similar between spleens from LPS90-exposed Wt and Het pups at PN12 compared with age-matched Wt pups delivered from untreated dams (controls) (Table 1). A significant decrease in splenic CD11b^+^Ly6G^+^ neutrophils was observed in LPS90-exposed Wt pups (19.53 ± 1.05) compared with Wt pups (23.77 ± 0.86) from age- and genotype-matched control moms (Table 1). Splenic CD11b^+^Ly6G^+^ neutrophils were not different between LPS90-exposed Wt and Het pups (Table 1). Significant increases in CD11b^+^CD11c^+^ (1.98 ± 0.11) and CD11b^+^CD11c^+^MHCII^+^ (1.92 ± 0.12) activated DCs in LPS90-exposed Wt pups were observed compared with Wt pups from age- and genotype-matched control moms. However, LPS90-exposed Het pups had significantly less CD11b^+^CD11c^+^ DCs (0.407 ± 0.022) (Table 1) and CD11b^+^CD11c^+^MHCII^+^ activated DCs (0.40 ± 0.03) (Table 1) compared with LPS90-exposed Wt pups. Splenic CD11b^+^Ly6C^+^ monocyte populations were similar between all groups (Table 1).

**Table 1:** Innate immune cell populations in spleens of Wt and Het offspring at PN12 from mothers treated with or without LPS at E15.5.

GROUP	AGE (n)	CD11b^+^Ly6G^+^	CD11b^+^CD11c^+^	CD11b^+^CD11c^+^MHCII^+^	CD11b^+^Ly6C^+^
		HO-1 (MFI)	HO-1 (MFI)	HO-1 (MFI)	HO-1 (MFI)
**Wt Control**	**PN12 (6)**	23.77 ± 0.86	0.617 ± 0.091	0.60 ± 0.090	5.19 ± 0.41
		1300 ± 67	2443 ± 164	2438 ± 167	2042 ± 112
**Wt-LPS90**	**PN12 (4)**	**19.53 ± 1.05**^¶^	**1.98 ± 0.11**^†^^§^	**1.92 ± 0.12**^†^^§^	5.64 ± 0.43
		1167 ± 12	1823 ± 43	1812 ± 38	1769 ± 27
**Het-LPS90**	**PN12 (3)**	21.80 ± 1.30	0.407 ± 0.022	0.40 ± 0.03	4.88 ± 0.16
		**3374 ± 359**^†^^‡^	**7160 ± 811**^†^^‡^	**7160 ± 811**^†^^‡^	**5979 ± 513**^†^^‡^

All values are expressed as mean ± SD. Innate immune cell populations expressed as %CD45^+^, HO-1 expression in MFI units.

^¶^ For CD11b^+^Ly6G^+^ Wt-LPS90 at PN12 compared with Wt Control at PN12 p = 0.0277;

^†^ Wt LPS90 PN12 compared with Wt Control PN12 (p<0.0001) for CD11b^+^CD11c^+^ and CD11b^+^CD11c^+^MHCII^+^;

^§^ Wt LPS90 PN12 compared with Het LPS90 PN12 (p<0.0001) for Het-LPS90 compared with CD11b^+^CD11c^+^ and CD11b^+^CD11c^+^MHCII^+^;

^†^ Wt control PN12 and

^‡^ Wt LPS90 at PN12 (p<0.0001) for CD11b^+^Ly6G^+^ HO-1 MFI, CD11b^+^CD11c^+^ HO-1 MFI, CD11b^+^CD11c^+^MHCII^+^ HO-1 MFI and CD11b^+^Ly6C^+^ HO-1 MFI.

Results in bold are significant.

### Intracellular HO-1 expression in innate immune cells (offspring spleen) at PN12 after maternal treatment with LPS

Intracellular HO-1 expression in splenic CD11b^+^Ly6G^+^ neutrophils (3374 ± 359) (Table 1), CD11b^+^CD11c^+^ DCs (7160 ± 811) (Table 1), CD11b^+^CD11c^+^MHCII^+^ activated DCs (7160 ± 811) (Table 1), and CD11b^+^Ly6C^+^ monocytes (5979 ± 513) (Table 1) were significantly increased in LPS90-exposed Het pups compared with pups from age-matched similarly treated Wt and Wt control moms.

### Innate immune responses and HO-1 in offspring at PN25 compared with PN12 after maternal treatment with LPS

**Fig 5:**
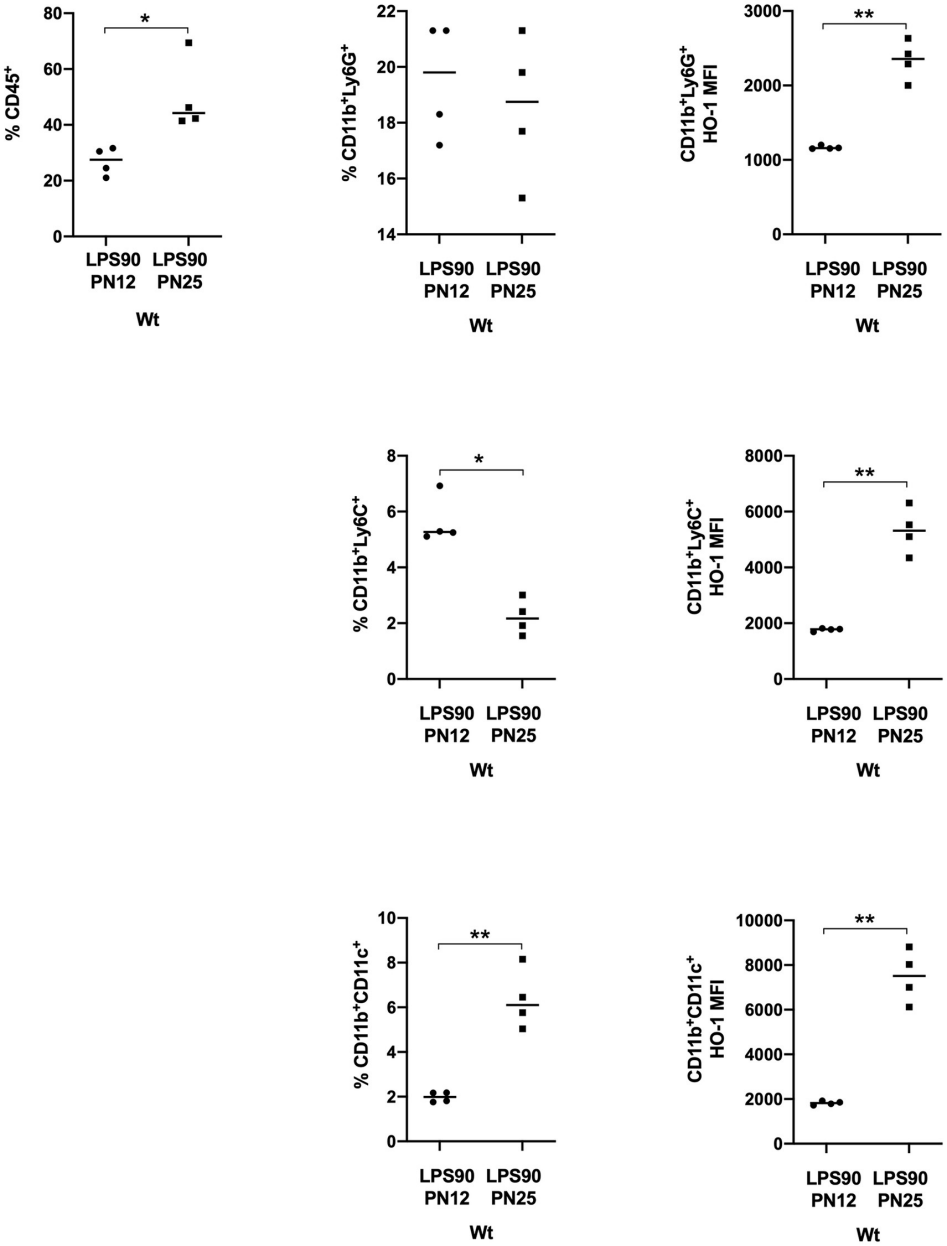
Innate immune responses at PN25 compared with PN12 in Wt offspring from dams exposed to LPS at a dose of 90 ug/kg (LPS90) late in gestation. LPS administered i.p. to pregnant Wt dams at E15.5. Expression of splenic innate immune cells and intracellular HO-1 in Wt offspring were determined at PN25 by flow cytometry (Wt LPS90 PN12, n = 4; Wt LPS90 PN25, n = 4). CD45^+^ common leukocyte antigen. CD11b^+^Ly6G^+^ neutrophils. CD11b^+^CD11c^+^DCs. CD11b^+^Ly6C^+^ (mononuclear cells). Intracellular HO-1 expression in neutrophils, DCs, and monocytes. Cell populations are shown as percentages of CD45^+^ cells and intracellular HO-1 expression as median fluorescent intensities (MFIs). Values are expressed as mean ± SD, p < 0.05, Student’s t-test with Welch correction.

Percentages of CD45^+^ cells and CD11b^+^CD11c^+^ DCs were significantly higher at PN25 compared with PN12 in LPS-exposed Wt pups. CD11b^+^Ly6C^+^ mononuclear cells were significantly decreased; whereas, CD11b^+^ Ly6G^+^ cells were unchanged at PN25 compared with PN12 after exposure to LPS in Wt pups. Intracellular HO-1 expression was significantly increased at PN25 compared with PN12 in neutrophils and mononuclear cells, but was similar in DCs at PN25 compared with PN12 in LPS-exposed Wt pups (Fig 5).

## Discussion

We observed that maternal inflammation late in gestation can induce alterations in fetal HO-1 expression and dysregulation of splenic innate immune cell populations in offspring. Here, we first assessed maternal and fetal well-being following a single maternal inflammatory insult late in gestation (at E15.5). Second, we determined subsequent alterations in maternal and fetal tissue HO activities compared with NP, age-matched adult females. Finally, we identified differential innate immune system changes in offspring of pregnant Wt and Het dams treated with LPS late in gestation.

Maternal exposure to LPS can cause adverse fetal and neonatal outcomes by altering the fetoplacental microenvironment [27]. The dose of LPS that induces an adverse effect can vary depending on the animal model, LPS strain, route of administration, and GA at exposure [28]. In maternal inflammatory response (MIR) models using LPS or fetal inflammatory response models (FIR) utilizing intra-amniotic inflammation, unique cytokine/chemokine patterns have been reported in the mother and the fetus [27]. Although both MIR and FIR models result in increased pro-inflammatory cytokines in the fetus, FIR is less pronounced after LPS is administered i.p. [27]. A maternal septic response with increased maternal mortality in mice, however, is not detected until very high doses of LPS (10 to 40 mg/kg) are given [29]. Our mouse model is similar to a late pregnancy MIR model, by not causing overt maternal morbidity or mortality. However, we do show that fetal and offspring health are negatively affected when dams are given LPS90 late in gestation. In a recent study, 20 hrs after treatment with 250 to 1000 μg/kg LPS i.p. at E13 to E17, detrimental effects on the fetus were observed to be most pronounced at E16 to 18 [30]. Using our model at an i.p. LPS dose of 250 μg/kg, we confirmed that at E16.5, there is a significant decrease in the percentage of live fetuses (43.5%) compared with GA-matched Wt controls, and at E18.5, there is no fetal survival (see S2 Fig). Therefore, we did not administer LPS250 to pregnant Het dams.

It is well known that Het/Het breeding pairs result in increased fetal mortality, placental insufficiency, and intrauterine growth restriction compared with Wt breeding in the absence of any inflammatory stimuli [31]. Similar to the original paper by Poss and Tonegawa [23], we observed a low fetal survival percentage. This is as expected since our HO-1-deficient mouse colony is derived from their original colony. We have also reported that placental vascular defects in pregnant HO-1-deficient dams may be mediated by changes in angiogenesis and vasculogenesis, which might contribute to a low fetal survival rate and appear as an “apparent” infertility [12, 31]. Our findings of decreased fetal survival in Het pregnancies are in agreement with previously published data [12, 15, 32]. We also have shown a similar rate of survival at delivery between Het and Wt offspring after treatment of pregnant mice with LPS90 at E15.5 [2]. In the current study, 24 hrs after treatment with LPS90 (at E16.5), survival in Wt and Het fetuses was similar and consistent with our previous survival to delivery results [2].

It is known that HO activity is an index of functional HO in a tissue [33]. In a recent study, Tsur et al. [15] measured baseline HO activity in untreated pregnant control dam tissues in Wt and Het FVB mice at E14.5. We found that liver, spleen, and placental HO activities of pregnant Wt controls were comparable with those we have previously reported [15], albeit our measurements were taken at E16.5, a slightly later timepoint in pregnancy. Additionally, we show that a LPS90 treatment at E15.5 does not change HO activity in pregnant Wt dam livers and spleens compared with NP and control pregnant dams, suggesting that LPS90 does not cause a significant maternal stress response in this model.

With regard to placental HO activity, we did not find a difference between LPS-treated and control dams. To our knowledge, there is no other study that has reported placental HO activity after exposure to LPS90 at E15.5. In contrast, placental HO-1 mRNA and protein levels have been more widely studied. Significant increases in placental HO-1 expression, 2 to 6 hrs after intrauterine LPS administration was found at E17.5 by RT-PCR in other perinatal inflammation models [34]. Future studies, focusing on placental HO-1 expression 2 to 6 hrs following LPS90 treatment at E15.5 would be important in determining differences in placental HO-1 expression in our model. In a separate study, 12 hrs after treatment of dams with LPS (75 μg/kg) i.p. at E17, placental HO-1 protein expression was significantly upregulated and was sustained for 48 hrs, albeit this dose did not result in fetal loss or preterm labor [35]. Furthermore, LPS-induced HO-1 upregulation in the placenta was significantly decreased by inhibition of TNF-α [35]. Previously in our model, we observed an upregulation of placental tumor necrosis factor receptor 1-alpha (Tnfrs1α) expression 24 hrs after LPS90 using PCR arrays (see S3 Fig). It is important to note that an increase in Tnfrs1α is involved in choriodecidual apoptosis and leads to uterine involution (placenta/myometrium) at the end of pregnancy [36]. Others have shown an increase in placental HO-1 mRNA and protein expression 4 hrs after treatment with 300-μg/kg LPS i.p. at E15 by RT-PCR and Western Blots [37]. However, none of these studies measured placental HO activity and hence cannot be compared directly with our placental HO activity findings.

Interestingly, 24 hrs after treatment with LPS90, we did detect an increase in HO activity in pooled liver/spleen samples suggesting an increase in fetal tissue stress response. However, we did not observe any correlation of dam HO activity and fetal HO activity. Others have reported significant increases in fetal liver HO-1 expression by Western Blots 24 hrs after pregnant mice were treated with 75 μg/kg LPS i.p. at E17 [38]. In that study, starting with 2 hrs after treatment, fetal liver HO-1 protein expression reached a maximum at 24 hrs and decreased to baseline at 48 hrs; whereas, lower LPS doses (< 75 μg/kg) did not result in any changes [38]. However, it is also important to note that upregulation in HO-1 mRNA may not translate to functional enzyme; and in that study, fetal organ HO enzyme activity was not measured [38]. Collectively, our results suggest that fetuses born to mothers exposed to inflammation late in gestation are able to mount a stress response via an upregulation of HO activity without significant increases in maternal HO activity and/or maternal illness.

With respect to fetal brain HO activity, we did not find differences between LPS-exposed and control fetuses. In the absence of inflammation, brain HO activity is developmentally regulated, being highest in the mature fetus [39]. However, the effect of inflammation on fetal brain HO activity has not been well-established. Thus, our results provide some insight into the potential effects of a low-grade maternal systemic inflammation late in pregnancy on fetal brain HO activity. However, it is known that the fetal brain cytokine response peaks at 2 to 12 hrs when pregnant dams are treated with a low dose LPS (50 μg/kg) at E18 [40]. Therefore, we believe studying earlier and later timepoints after maternal inflammation could be more informative in determining whether any differences in fetal brain HO activity occurs.

It is also known that HO-1 is particularly important for the immune system [1, 13]. Neutrophils, DCs, and monocytes are key antigen presenting cells (APCs) of the innate immune system, which in turn can prime T-cells for mounting adaptive immune responses [1, 41]. The maturational state of APCs is regulated by HO-1, which determines whether they go on to initiate antigen presentation to T cells [1, 13]. Neonatal neutrophils are known to be functionally impaired [27]. In our current study, we investigated two developmentally important critical timepoints in the immune system, PN12, which is the mouse equivalent to human childhood, and PN25, the mouse equivalent to young human adulthood [2, 42]. At PN12, the percentage of neutrophils decreased in LPS-exposed Wt pups compared with Wt controls without a detectable change in intracellular HO-1 expression. In Hets, the percentage of neutrophils is not different compared with that of LPS-exposed Wt pups, but showed an increase in intracellular HO-1 expression. An upregulation of HO-1 in neutrophils has been reported to be associated with an increased oxidative burst and in certain instances correlated with disease severity [43], but also associated with impaired neutrophil function [44]. Therefore, in Het pups from dams treated with LPS late in gestation, further studies to determine the functional status of neutrophils would be important.

In our model, the response of DCs was different between Wt and Het pups at PN12 born from mothers treated with LPS. Among all innate immune cells, DCs are the most potent APCs, and responsible for initiating adaptive immune system responses. From a simplistic view, during a typical immune response to an inflammatory stimulus, DCs would switch from an immature to a mature phenotype, become activated, and prime T cells to generate pro- or anti-inflammatory responses [45]. In Wt offspring from dams treated with LPS late in gestation, the percentage of MHCII^+^ DCs was increased, but intracellular HO-1 expression was not significantly altered compared with age- and genotype-matched controls at PN12. This contrasted starkly with our findings in Het offspring where we detected a significant increase in intracellular HO-1 expression at PN12 despite no detectable changes in the percentage of splenic DCs compared with Wt control pups and Wt pups born to mothers treated with LPS. This is not surprising since we previously reported differences in adaptive immune system responses in Wt and Het offspring and these alterations were more pronounced in Het offspring [2].

The effects of HO-1 on the monocyte phenotype and function are more complex as its upregulation can be associated with pro- or anti-inflammatory responses based on the microenvironment and disease model [46]. Mononuclear cells upon stimulation can polarize to either a pro-inflammatory M1 or an anti-inflammatory M2; however, this view is a little too simplistic, as there are other subtypes such as oxidative-macrophages [47]. These M2 and oxidative macrophages express HO-1 highly [47]. The HO-1 expression level in macrophages in a liver ischemia-reperfusion injury model correlates with a favorable macrophage phenotypic polarization to M2 [48]. However oxidative macrophages may be damaging. At PN12, percentages of monocytes were comparable in LPS-exposed Wt, Wt control, and LPS-exposed Het pups. LPS-exposed Het pups had an elevated intracellular HO-1 expression when compared with similarly treated age-matched Wt pups and Wt controls. It is important to note that, LPS-exposed Wt pups showed a decrease in monocytes and further increases in DCs at PN25 compared with PN12, and intracellular HO-1 expression was higher in neutrophils and monocytes at PN25 compared with PN12. This may suggest either developmental differences and/or effect of maternal inflammation late in gestation. It has been shown that a total deficiency of HO-1 (HO-1^-/-^) results in mononuclear cell (macrophage) dysfunction [49]. Furthermore, HO-1 knockout animals also have a pro-inflammatory predisposition, splenocytes isolated from these animals have a higher proinflammatory cytokine profile after *in vitro* LPS stimulation despite having similar proportion of mononuclear cells when compared with their Wt counterparts [50].

It is important to note that HO-1 expression in humans is genetically variable due to polymorphisms in the number of (GT)n dinucleotide repeats in the HO-1 promoter region or HO-1 mutant alleles [1]. Long (GT)n repeats is associated with a decrease in HO-1 gene expression [1] and may affect the inflammatory response in certain disease states, such as inflammatory vascular diseases (e.g., atherosclerosis) [51] and cardiovascular diseases [51]. These adverse vascular effects can be extrapolated to pathologic pregnancies. In fact, we have previously shown that a deficiency in HO-1 negatively effects maternal-fetal hemodynamics, placental angiogenesis, placental immune cell distribution, and also impacts fetal growth [10–12, 31, 52]. Furthermore, in an *in vitro* study, Taha *et al* [53] showed that HO-1 promotor polymorphisms can affect basal and induced HO-1 expressions using human umbilical vein endothelial cells (HUVEC) obtained from term neonates at birth. They reported that short (GT)n repeats were not only associated with high HO-1 expression; but also, with less production of pro-inflammatory mediators when exposed to oxidant stress [53]. Further studies are warranted to assess the role of HO-1 promoter polymorphisms in pregnant mothers in regard to their inflammatory response in late gestation and their potential development of pregnancy complications and adverse neonatal sequelae.

Our findings suggest that Wt pups regulate their immune system and HO-1 homeostasis more readily than Het pups born from mothers exposed to inflammation late in gestation. In fact, basal HO-1 levels are more important in determining whether there will be recovery or sustained injury after an insult [22]. Hence, the low baseline HO-1 expression in Hets may be a risk factor for a sustained dysregulation of the innate immune system. Furthermore, it is well known that an exaggerated expression of HO-1 can readily negate its protective effects [16].

Upregulation of intracellular HO-1 expression in neutrophils, DCs, and monocytes of LPS-exposed Het pups may suggest that because Het cells are in an unbalanced oxidoreductive environment, they have a heightened PN stress response even in the absence of a second hit. For example, differentiation of mononuclear cells from early progenitors to functional innate immune cells (macrophages) requires HO-1; and deficiency of HO-1 results in arrest in differentiation of macrophages [13]. HO-1 in inflammatory conditions can decrease neutrophil infiltration [13]. Additionally, deficiency of HO-1 results in sustained activation in APCs and inhibition of HO activity results in a heightened intracellular oxidative environment and pro-inflammatory cytokines [13]. In contrast APCs that express HO-1 have an anti-inflammatory cytokine secretion pattern and do not acquire an intracellular oxidative milieu [13]. These unique derangements in APCs and intracellular HO-1 expression patterns may account for our observed persistent adaptive immune system alterations [2]. Thus, the functional consequences of increased HO-1 expression in APCs on immune system reprograming in Het pups will require further study.

Our data show that exposure to an inflammatory stressor during gestation has differential effects on composition, percentages, and behavior of innate immune cells in Het compared with Wt offspring. This suggests that LPS-exposed Het pups were disproportionately affected as shown by the significant upregulation of intracellular HO-1 expression in splenic innate immune cells (neutrophils, DCs, and monocytes) compared with similarly treated Wt offspring and Wt controls at PN12. In contrast, innate immune cells in LPS-exposed Wt offspring appear to mount a similar upregulation of intracellular HO-1 at PN25, but not at PN12. We thus speculate that our data might suggest PN maturational differences between Het and Wt innate immune cells. Because Het cells under-express HO-1 at baseline [21], this appears to be paradoxical. It does however suggest that Het cells have an exaggerated and earlier oxidative stress response, which may be due to the pro-inflammatory predisposition of the Het phenotype. Moreover, it has also been shown that the degree of HO-1 upregulation correlates with organ damage [21]. Thus, our observed increase in intracellular HO-1 in cells from LPS-exposed Het pups at PN12 as opposed to PN25 in LPS-exposed Wt pups may suggest a greater degree of inflammation and immune organ injury in Het cells at an earlier PN age compared with Wt cells.

Our study is relevant to the understanding of pathologies associated with placental vascular development, placental insufficiency, and/or altered placental immune infiltrates [1, 11, 12, 31, 52], especially in terms of infections occurring late in gestation and how they may adversely affect fetal/neonatal outcomes [1, 2]. Future studies utilizing human maternal and neonatal samples stratified by the presence of HO-1 polymorphisms in healthy pregnancies and pregnancies complicated with infection or inflammation may lead to a better understanding of how maternal and fetal HO-1 expression impacts neonatal outcomes.

There are however some limitations to our study. We did not measure maternal liver, spleen, and placental HO activities or fetal tissue HO activity in Het mice, but we have previously reported these activities to be significantly lower compared with Wt tissues at E14.5 [15]. In addition, we did not measure liver HO activity at our PN timepoints; but again, HO activity has been previously shown to be comparable between 1-wk-old Wt and Het offspring [25]. Nonetheless, we do show an increased HO-1 expression in certain immune cells of Het pups compared with those in Wt pups. We attribute this dysregulated pro-inflammatory baseline to PN maturational differences between LPS-exposed Het and Wt innate immune cells. However, this may also reflect the known vulnerability of Het animals to stressors with their pro-inflammatory predisposition. Although our findings add to the literature describing the phenotype of offspring of Het mothers treated with LPS, they do not address the possible impact of late exposure of the fetus to inflammation independent of the Het phenotype. Therefore, what we can only conclude is that the offspring of Het mothers exposed to LPS late in gestation in this model have dysregulation of their innate immune system, distinguishing them from Wt offspring of Wt mothers treated in the same way. Whether offspring of Het mothers exposed to LPS late in gestation are different from Het offspring whose mothers were not exposed to LPS late in gestation remains a question deserving of study. Future studies are proposed that may elucidate this mechanism by focusing on Het animals and other PN timepoints.

## Conclusions

In summary, we have demonstrated that maternal inflammation late in gestation can induce alterations in maternal-fetal HO-1 expression and lead to a dysregulation of the innate immune system in offspring. Most interestingly, we also have shown a dysregulation of intracellular HO-1 expression in myeloid cells in LPS-exposed Het pups. We speculate that the upregulation of intracellular HO-1 in Het APCs, the persistent innate immune system activation with unfavorable immune cell phenotypes, and the inherently low basal HO-1 levels in Het offspring places them to an increased risk for developing future immune-mediated diseases.

## Supporting information

S1 Fig A representative gating strategy for the flow cytometry studies is shown. Debris and doublets were excluded by sequential gating on forward scatter height versus forward scatter area. Anti-CD19-Pacific Blue and anti-CD3-γΔTCR were used as dump channels. After gating for viable cells, we sequentially gated for CD45 (leukocytes), CD45 CD11b CD11c (dendritic cells [DCs]), CD45 CD11b Ly6G (neutrophils), and CD45 Ly6C (mononuclear cells) populations.
(TIFF)

S2 Fig Percent live fetuses after LPS90 and LPS250 compared with Wt controls. Intraperitoneal administration of LPS at a dose of 250 μg/kg at E16.5 resulted in a significant decrease in the percentage of live fetuses compared with GA-matched Wt controls. At E18.5, we observed no fetal survival. E16.5 Control (n = 9) vs. E16.5 LPS250 (n = 4), **p = 0.0072; E16.5 LPS90 (n = 8) vs. E18.5 LPS250 (n = 1), **p = 0.0018; E16.5 Control (n = 9) vs. E18.5 LPS250 (n = 1), ***p = 0.0006.
(TIFF)

S3 Fig Expression of immune genes in Wt placentas 24 hrs after LPS90 treatment. Using PCR arrays, we observed a significant upregulation of tumor necrosis factor receptor 1-alpha (Tnfrs1α) expression in Wt placentas (n = 3) 24 hrs after LPS90 compared with Wt control placentas (n = 3, p = 0.021).
(TIFF)

S1 Table Body weights of Wt and Het dams with or without LPS treatment at E15.5 and E16.5. LPS90 was administered i.p., at E15.5 to pregnant (P) Wt and Het dams. LPS90-treated Wt dams did not lose weight at E16.5 (n = 18) compared with age-matched pregnant Wt dams (n = 6). LPS90-treated Het dams did not lose weight at E16.5 (n = 8) compared with age-matched pregnant Het dams (n = 7 for both embryonic ages). p > 0.05.
(DOCX)
