# Supplementary figures and images for: Inflammation-induced alterations in maternal-fetal Heme Oxygenase (HO) are associated with sustained innate immune cell dysregulation in mouse offspring
Source: PLoS One. 2021 Jun 4;16(6):e0252642. doi: 10.1371/journal.pone.0252642 (PMC8177474; doi:10.1371/journal.pone.0252642)

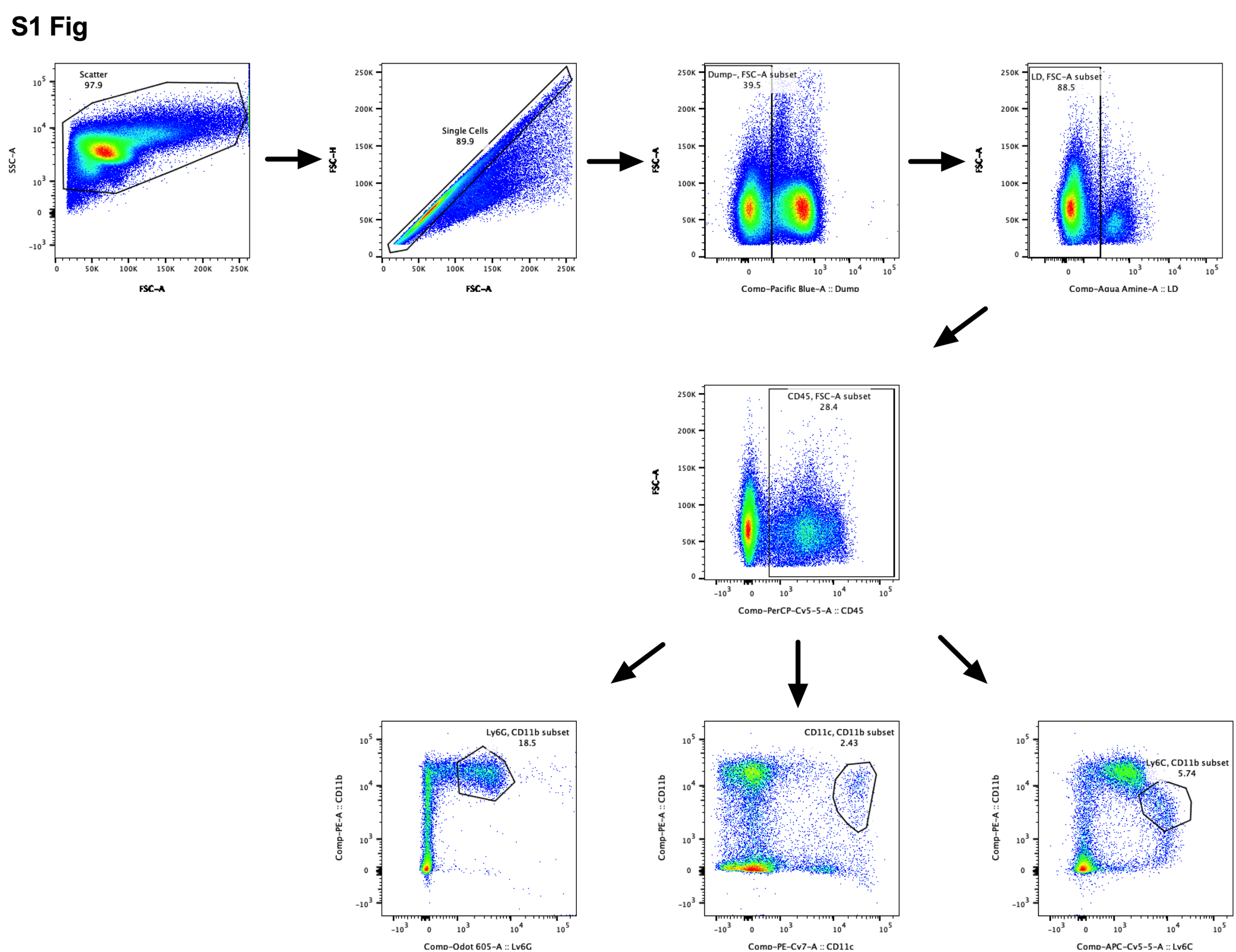

Supplement: S1 Fig — Debris and doublets were excluded by sequential gating on forward scatter height versus forward scatter area. Anti-CD19-Pacific Blue and anti-CD3-γΔTCR were used as dump channels. After gating for viable cells, we sequentially gated for CD45 (leukocytes), CD45 CD11b CD11c (dendritic cells [DCs]), CD45 CD11b Ly6G (neutrophils), and CD45 Ly6C (mononuclear cells) populations. (TIFF) [file pone.0252642.s001.tiff]

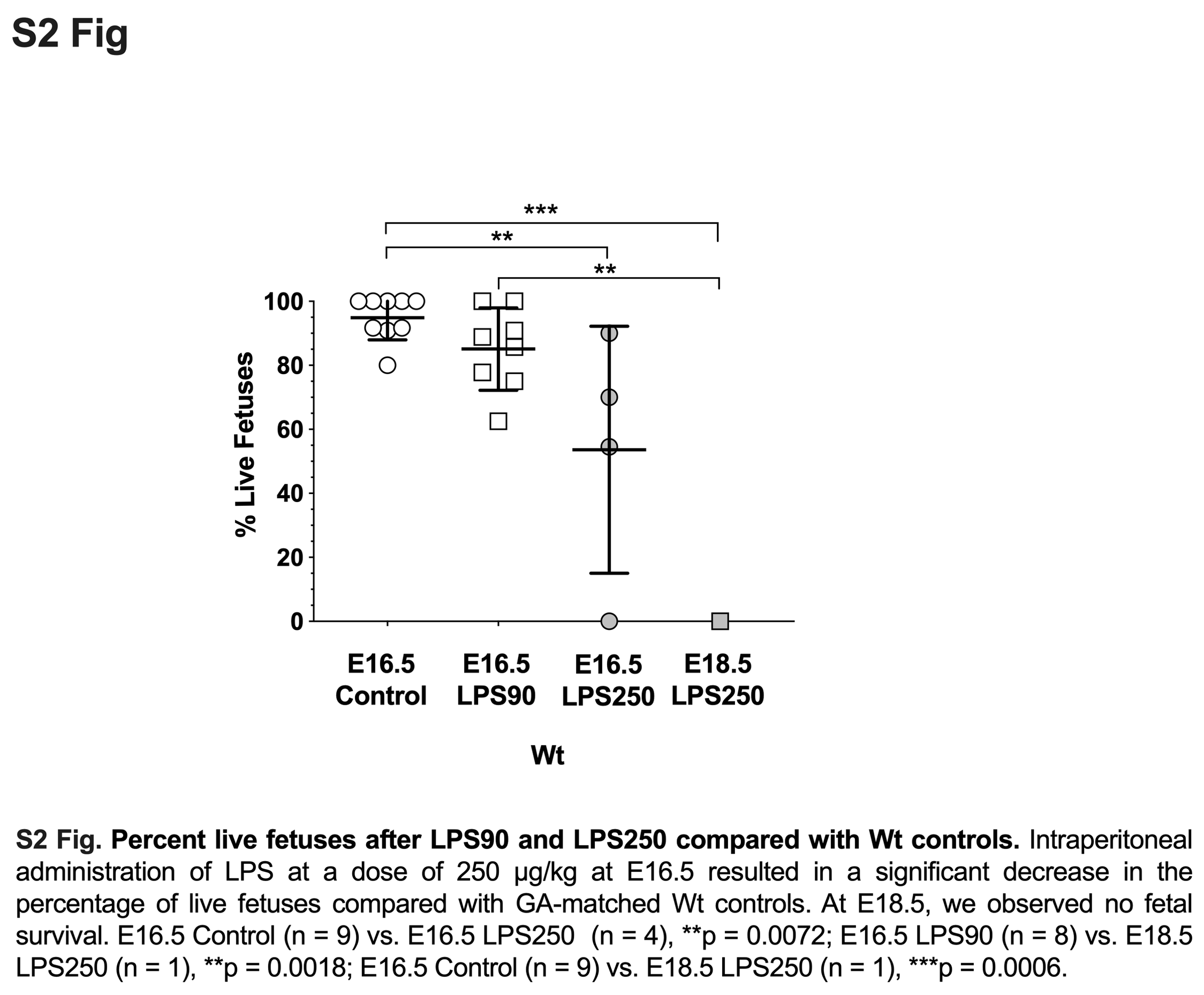

Supplement: S2 Fig — Intraperitoneal administration of LPS at a dose of 250 μg/kg at E16.5 resulted in a significant decrease in the percentage of live fetuses compared with GA-matched Wt controls. At E18.5, we observed no fetal survival. E16.5 Control (n = 9) vs. E16.5 LPS250 (n = 4), **p = 0.0072; E16.5 LPS90 (n = 8) vs. E18.5 LPS250 (n = 1), **p = 0.0018; E16.5 Control (n = 9) vs. E18.5 LPS250 (n = 1), ***p = 0.0006. (TIFF) [file pone.0252642.s002.tiff]

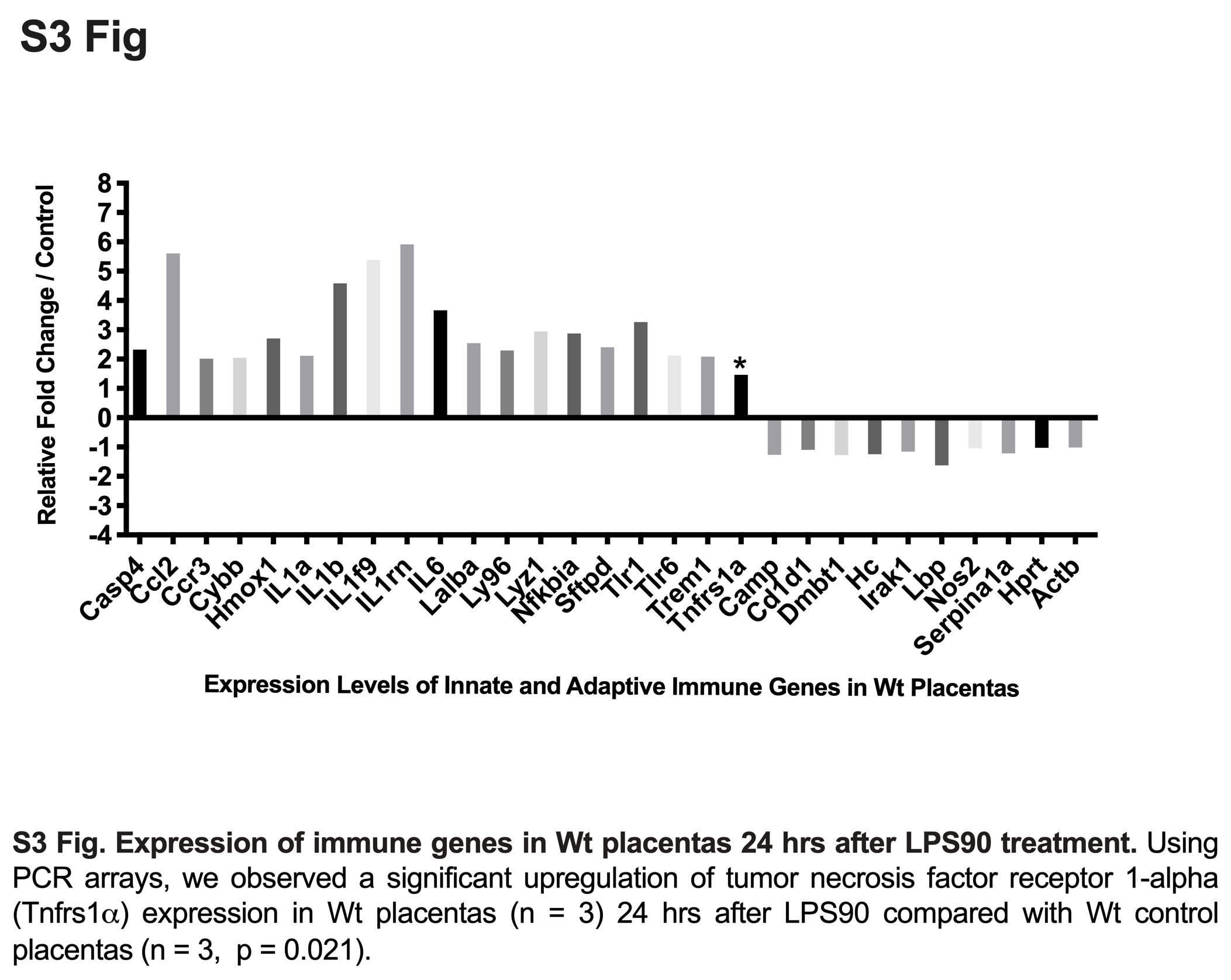

Supplement: S3 Fig — Using PCR arrays, we observed a significant upregulation of tumor necrosis factor receptor 1-alpha (Tnfrs1α) expression in Wt placentas (n = 3) 24 hrs after LPS90 compared with Wt control placentas (n = 3, p = 0.021). (TIFF) [file pone.0252642.s003.tiff]
