# Supplementary material for: Inflammation-induced alterations in maternal-fetal Heme Oxygenase (HO) are associated with sustained innate immune cell dysregulation in mouse offspring
Source: PLoS One. 2021 Jun 4;16(6):e0252642. doi: 10.1371/journal.pone.0252642 (PMC8177474; doi:10.1371/journal.pone.0252642)
Supplement: S1 Table — LPS90 was administered i.p., at E15.5 to pregnant (P) Wt and Het dams. LPS90-treated Wt dams did not lose weight at E16.5 (n = 18) compared with age-matched pregnant Wt dams (n = 6). LPS90-treated Het dams did not lose weight at E16.5 (n = 8) compared with age-matched pregnant Het dams (n = 7 for both embryonic ages). p > 0.05. (DOCX) [file pone.0252642.s004.docx]

**S1 Table**. **Body weights of Wt and Het dams with or without LPS treatment at E15.5 and E16.5**

| **Dam weight (g)** | **Wt** | | **Het** | |
| --- | --- | --- | --- | --- |
|  | **E15.5** | **E16.5** | **E15.5** | **E16.5** |
| **Control** | 34.8±2.8 | 35.6±2.3 | 34.6±3.8 | 36.4±3.4 |
| **LPS90** | 37.8±2.8 | 37.8±2.9 | 37.1±4.6 | 36.0±5.5 |

LPS90 was administered i.p., at E15.5 to pregnant (P) Wt and Het dams. LPS90-treated Wt dams did not lose weight at E16.5 (n = 18) compared with age-matched pregnant Wt dams (n = 6). LPS90-treated Het dams did not lose weight at E16.5 (n = 8) compared with age-matched pregnant Het dams (n = 7 for both embryonic ages). p > 0.05.
